# Characterization of chromatin regulators in hepatocellular carcinoma to guide clinical therapy

**DOI:** 10.3389/fgene.2022.961018

**Published:** 2022-08-25

**Authors:** Xiangen Jia, Guozhi Zhang

**Affiliations:** ^1^ North China University of Science and Technology Affiliated Hospital, Tangshan, China; ^2^ North China University of Science and Technology, Tangshan, China

**Keywords:** chromatin regulators, hepatocellular carcinoma, Signature, TCGA, ICGC

## Abstract

**Background:** Hepatocellular carcinoma (HCC) is notorious for its high mortality and incidence. Accumulating evidence confirms that chromatin regulators (CRs) have a significant impact on cancer. Therefore, exploring the mode of action and prognostic value of CRs is imminent for the treatment of hepatocellular carcinoma.

**Method:** Transcriptome and clinical data for this study have been downloaded from TCGA (https://portal.gdc.cancer.gov/) and ICGC (https://dcc.icgc.org/). Univariate analysis was used to screen CRs with prognostic value, and our prognostic risk score signature was developed using least absolute shrinkage along with selection operator (lasso) Cox regression analysis. The CRs-based prognostic model was constructed in the TCGA dataset, and low-risk HCC patients had a better prognosis, which was finally validated in the ICGC dataset. We used the receiver operating characteristic curve to identify the accuracy of the prediction model and establish a line chart to prove the clinical effectiveness of the model. We also discussed the differences in drug sensitivity *via* CellMiner database, tumor immune microenvironment *via* ssGSEA algorithm, and clinical characteristics among different risk groups.

**Results:** A prognostic model consisting of seven CRs was constructed and verified in HCC patients. Furthermore, we found that this risk score prognostic signature could independently predict the prognosis of HCC patients. Functional enrichment analysis revealed that CRs are mainly associated with cancer-related signaling pathways and metabolic pathways. In addition, immune cell abundance correlates with risk score levels

**Conclusion:** In brief, we systematically explored the mode of action of CRs in HCC patients and established a reliable prognostic prediction model.

## Introduction

Liver cancer is one of the highest incidences of cancer as well as the main cause of cancer-related death (Villanueva, 2019). Hepatocellular carcinoma is the main component of primary liver cancer, and its burden is increasing as a highly heterogeneous disease (Hoshida, Nijman et al., 2009; El-Serag, 2011; Nault and Villanueva, 2015). Despite advances in treatment strategies in recent years, overall survival in hepatocellular carcinoma remains disappointing (Njei, Rotman et al., 2015). Therefore, this study aimed to build a chromatin regulators model to predict overall survival in HCC samples to improve diagnostic accuracy and treatment efficacy.

Epigenetic alterations occur in through various forms, including methylation and histone modifications (Zhang, Lu et al., 2020). For example, CG14906 mediates M6A methylation of snRNA in *Drosophila melanogaster* (Gu, Wang et al., 2020). Epigenetic changes are associated with various diseases. Carbon 5 methylation of cytosine bases in the context of CpG dinucleotides involved in the onset (Wang, Gu et al., 2013) and progression of human diseases and enhanced commuting targeting c-Jun N-terminal kinase 2 (JNK2) epigenetic dysregulation of children is associated with impaired lung function in early childhood (Bauer, Trump et al., 2016). Among them, STAG2 regulates interferon signaling in melanoma through enhancer loop reprogramming (Chu et al., 2022), while BAZ2A is involved in epigenetic alterations in prostate cancer, and its overexpression predicts disease recurrence (Gu, Frommel et al., 2015). In addition, chromatin state also affects epigenetic changes (Blanco, Sykes et al., 2021). In recent years, advances in computer science techniques have brought new opportunities for cancer research, allowing us to sense molecular differences in disease using bioinformatics methods such as machine learning (Gu, Guo et al., 2020).

Chromatin regulators link the scale of chromatin organization from nucleosome assembly to the establishment of functional chromatin domains. For example, centromeres have a unique histone variant, CenH3, that marks the site of motor body assembly and microtubule attachment required for proper chromosome segregation (Cleveland, Mao et al., 2003). Epigenetic change also is one of the most important factors in tumor, and CRs are an indispensable regulatory element to drive this process (Lu, Xu et al., 2018). CRs are mainly divided into three categories: DNA methylates, histone modifiers, and chromatin remodelers, and these CRs are inseparable and function together in biological processes (Plass, Pfister et al., 2013). Previous studies have shown that mutant chromatin regulatory factors are the driving factor of cancer (Gonzalez-Perez, Jene-Sanz et al., 2013; Koschmann, Nunez et al., 2017), which suggests that dysregulation of chromatin regulators is closely related to cancer (Marazzi, Greenbaum et al., 2018). In addition, mutations in chromatin regulators, such as ARID1A, ARID1B, ARID2, MLL, and MLL3, can also cause liver cancer (Fujimoto, Totoki et al., 2012). ASCL2, a chromatin regulator, is upregulated in colorectal cancer cells, and its downregulation enhances autophagy to promote apoptosis in colorectal cancer cells (Marazzi, Greenbaum et al., 2018). As a member of chromatin regulators, FTO can increase the response to chemotherapy drugs through demethylation of colorectal cancer cells (Relier, Ripoll et al., 2021). WHSC1 regulates BCL2 expression and apoptosis in HCC, elucidating a novel epigenetic regulation mechanism (Wang, Zhu et al., 2021). However, few studies have systematically and comprehensively investigated the role of CRs in HCC.

In this study, we systematically studied the expression profile of CRs in HCC and its prognostic value. We successfully developed and demonstrated that the prognostic signature derived from seven CRs could be used to predict the prognosis of patients with HCC. In addition, we also proved that the prognostic signature can accurately predict the immune microenvironment of tumors, which may provide a foundation for future immunotherapy strategies.

## Materials and methods

### Data acquisition and differential expression analysis

Transcriptomic data for 50 normal liver tissues and 374 HCC samples and clinical data for 377 HCC samples were downloaded from the TCGA database. Gene expression profiles were normalized, and differentially expressed genes (DEGs) were also analyzed using the “limma” R package according to |fold-change| >1 and *p* < 0.05. Transcriptomic data and clinical data for 273 HCC samples were downloaded from the ICGC database (https://dcc.icgc.org/projects/LIRI-JP). Data from both the TCGA database and ICGC database were publicly available, and this study was exempt from local ethics committee approval.

A total of 870 chromatin regulators (CRs) were downloaded from the previous literature; the specific information is in Supplementary Table S1.

### Functional enrichment analysis

The biological function of CRs was explored using Gene Ontology (GO) analysis and the Kyoto Encyclopedia of Genes and Genomes (KEGG) pathway. *p* < 0.05 were set as significant thresholds, which were obtained using the “clusterprofiler” R package. The visualization of the enrichment results was then performed using the “enrichplot” and “ggplot2” R packages.

### Developing and validating the model

In this study, the TCGA data set was used as a training group, and the ICGC data set was used as a testing group. Univariate Cox regression analysis was used to screen CRs related to prognosis in the TCGA set. The “GLMNET” R package was used to analyze the prognosis-related CRs by LASSOCox regression to create a prognostic risk score model (Tibshirani 1997). The risk score was calculated in the following way:

Riskscore=∑(Expi∗coefi)

Based on the median risk score, the TCGA set and the ICGC set were divided into two groups: low-risk and high-risk; afterward, the survival analysis, the receiver operating characteristic (ROC) curve using the “survivalROC” R package (Blanche, Dartigues et al., 2013), and the principal component analysis (PCA) were conducted to understand the difference in transcriptional profiles between the low-risk and high-risk scoring groups. The “Rtsne” R package was used to perform t-distributed Stochastic Neighbor Embedding (t-SNE) analysis. The visualization of result was performed using the “ggplot2” R package.

### Correlation between risk signature and clinical characteristics

In the TCGA queue, the “CMScaller” R package was used to classify all samples as CMS. Each sample was combined with the clinical features from the TCGA cohort. Using the “limma” R package, we explored the relationship between risk score, gender, grade stage, age, pathological stage, TNM stage, and immune checkpoint in the TCGA cohort. The relationship between risk score and gender, age, and pathological stage was explored in the ICGC cohort.

### GSVA

Gene set enrichment analysis (GSVA) is a method that can evaluate biological processes by expressing matrix samples (Hänzelmann, Castelo et al., 2013). We used the dataset “C2.cp.kegg.v7.4” from MSIGDB (https://www.gsea-msigdb.org/gsea/msigdb) as a reference. The difference in biological processes between high and low-risk groups was explored using GSVA. FDR<0.05 showed that the biological pathway has statistical significance.

### Tumor immune correlation analysis

The ssGSEA method was used to explore the correlation between chromatin regulators and immune infiltration (Charoentong, Finotello et al., 2017). Immune cell abundance and immune-related functions were assessed for each sample in the TCGA database and ICGC database, and the differential analysis of immune score and immunophenotype was performed using the “Limma” R package. Spearman-related tests were then used to identify the relationship between risk score and immune checkpoint expression, stem cell index. Finally, we analyzed the immune score and matrix score of HCC samples and used the “estimate” R package and “Limma” R package to derive the scatter plot.

### Nomogram construction

Outcome-related nomograms were established using clinical variables and CRs-based risk scores to assess OS in patients with HCC using the “rms” R package. For the purpose of evaluating the prediction effect of a nomogram, consistency index (C index) and calibration curve were used. Then, a multivariate Cox regression analysis was carried out to explore whether risk score and clinical features have independent predictive value. AUC was calculated using the ROC curve to show the prognostic effect of the nomogram.

### Drug sensitivity analysis

Drug sensitivity data were collected from the CellMiner database to explore the drug sensitivity. Then, the relationship between the genes that make up the model and drug sensitivity was determined using the Pearson correlation test.

### Statistical analysis

The Wilcoxon rank sum test was used to compare the differences between the two groups. Kruskal–Wallis (KW) test for comparing three or more groups. All statistical analyses were performed in version R 4.1.2(*p* < 0.05).

## Results

### Enrichment analysis

Comparing the expression levels of chromatin regulatory factors in normal liver tissues and HCC tissues, 427 differentially expressed CRs were found in the TCGA cohort. *p* < 0.05 and |fold-change| >1 were set as the significance standard. In tumor tissues, the expression levels of 421 genes increased and 6 genes decreased. Supplementary Figure S1A shows the expression of differentially expressed CRs in normal and cancer tissues, which can be clearly distinguished. Then, GO and KEGG enrichment analysis was carried out on the differentially expressed CRs, respectively. Histone modification, chromatin organization, and peptidyl–lysine modification processes are highly enriched GO terms (Supplementary Figure S1B). Cell cycle, lysine degradation, and hepatocellular carcinoma are highly enriched KEGG terms (Supplementary Figure S1C), which indicates that chromatin regulators are indispensable in the development of hepatocellular carcinoma.

### Development of prognostic risk scoring signature in the training set

**FIGURE 1:**
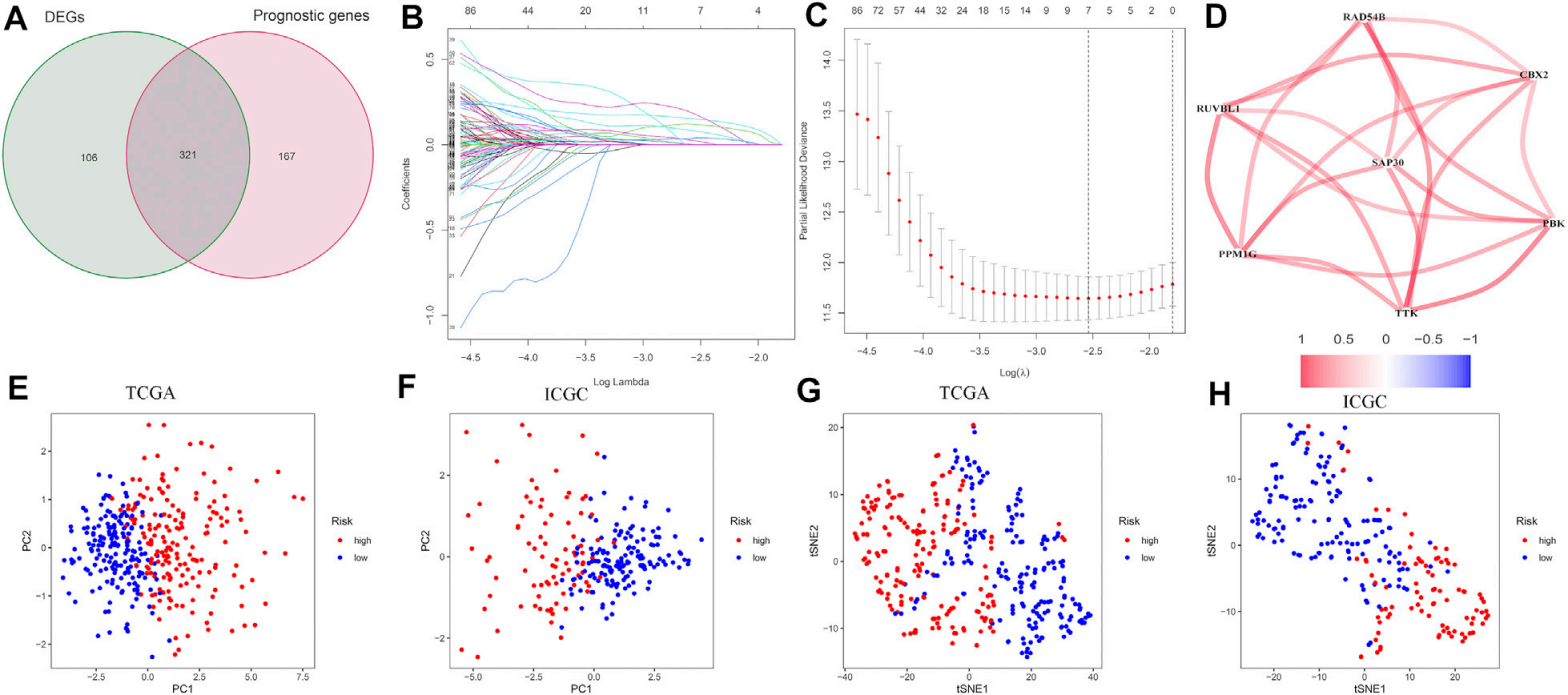
Development of the CR-based prognostic risk score model in the training set. **(A)** Venn diagram for tumor and normal tissue adjacent to carcinoma of differentially expressed genes related to the OS. **(B)** LASSO coefficients of seven CRs. **(C)** Identification of CRs for developing prognostic risk scoring models. **(D)** Correlation network of the CRs that makes up the model. Red lines indicate a positive correlation. **(E–H)** PCA and t-SNE assays based on chromatin regulator risk score to distinguish tumors from normal samples in the TCGA cohort and ICGC cohort. The low-risk patients are marked with green groups, and the high-risk patients are marked with red groups.

The construction of the prognostic model was performed in the TCGA set. The CRs associated with the prognosis of hepatocellular carcinoma was screened using univariate COX regression analysis. Then, the differential expression of CRs and prognosis-related CRs were intersected, and a total of 321 CRs were identified (Figure 1A). Then, we built the signature using lasso regression analysis. The final prognostic risk score model consists of seven genes (genes CBX2, PBX, PPM1G, RAD54B, RUVBL1, SAP30, and TTK) (Figures 1B and C). The risk score signature is constructed as follows: risk score = (expression of CBX2*0.149956574108953) + (expression of PBK*0.000160217 695501227) + (expression of PPM1G*0.118132434310044) + (expression of RAD54B*0.175317229614019) + (expression of RUVBL1*0.0794852407313874) + (expression of SAP30*0.02236 60675255999) + (expression of TTK*0.039488102659109). The correlations between the genes that make up the risk scoring model are shown in Figure 1D. PCA and t-SNE analysis show identifiable dimensions between the low-risk score group and high-risk score group (Figures 1E–H).

### Clinical correlation analysis of risk scoring signature in TCGA database

**FIGURE 2:**
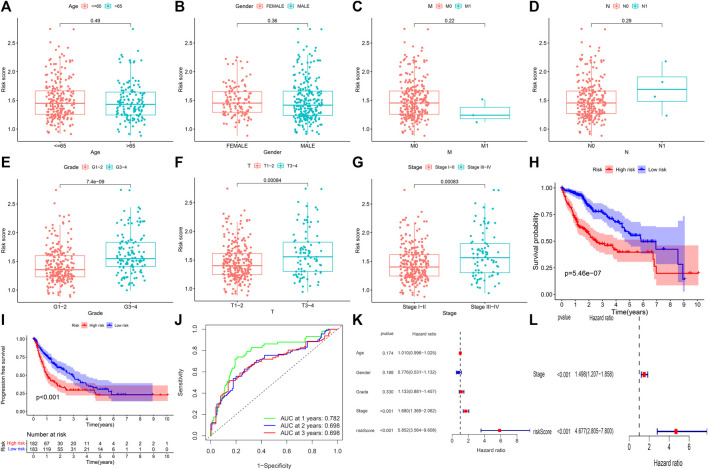
Predictive value of CRs scoring model for the survival status of HCC patients in TCGA queue. **(A–G)** The relationship between risk score and clinicopathological factors, including age **(A)**, gender **(B)**, distant infiltration **(C)**, lymphoid metastasis **(D)**, grade stage **(E)**, tumor infiltration **(F)**, and pathological stage **(G)**. **(H** and **I)** Comparison of total survival time (OS) and progression-free survival (PFS) in low-risk and high-risk groups. **(J)** Sensitivity and specificity of risk scores measured by ROC curves for predicting 1-, 3-, and 5-year overall survival. **(K** and **L)** Forest diagram of univariate and multivariate Cox regression analyses.

**FIGURE 3:**
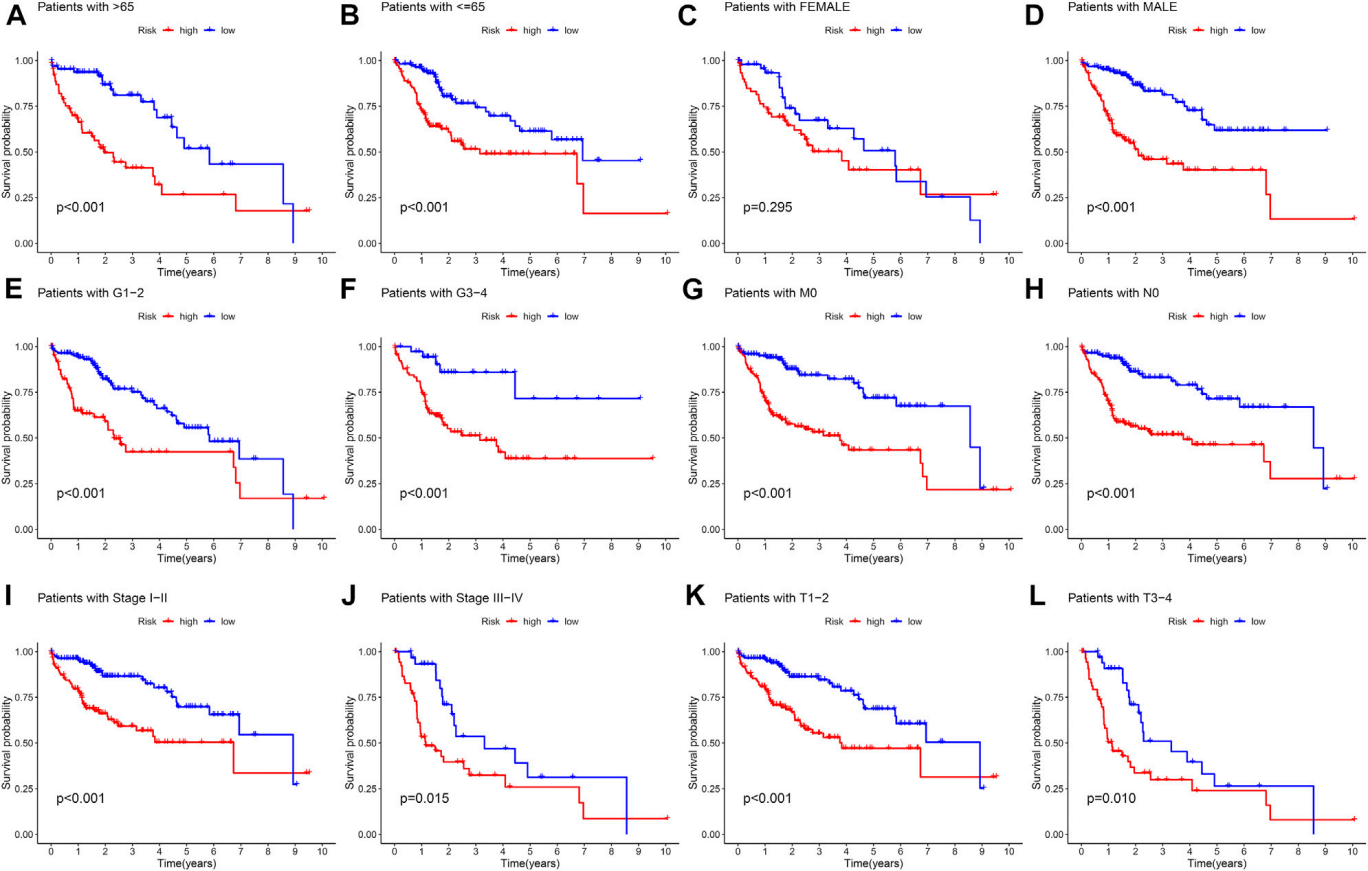
Kaplan–Meier curves of OS differences stratified by sex, age, grade, pathological stage, N stage, M stage, and T stage between high-risk and low-risk groups. **(A,B)** Age >65 and Age<=65. **(C,D)** Female and male. **(E,F)** Grade stage 1–2 and Grade stage 3–4. **(G,H)** M0 stage and N0 stage. **(I,J)** pathological stage I–II and pathological stage III–IV. **(K,L)** T stage 1–2 and T stage 3–4.

The samples in the training set were divided into low (183) and high (182) risk score groups according to the mean risk score. The relationship between clinical characteristics (age, gender, grade, pathological stage, and TMN stage) and risk score in the TCGA group was further analyzed. Risk score distribution was not statistically different between the TCGA groups with respect to age, gender, M stage (distant metastasis), and N stage (lymphatic metastasis) (Figures 2A–D). Higher risk scores were associated with higher grade stage (*p* = 7.4e-09; Figure 2E), higher T (tumor infiltration) stage (*p* = 0.00084; Figure 2F), advanced pathological stage (*p* = 0.00083; Figure 2G), poor overall survival (*p* = 5.46e-07; Figure 2H), and poor progression-free survival (PFS) (*p* = 5.46e-07; Figure 2I) in the training set. Figure 2H shows that the high-risk group had a significantly higher mortality rate than the low-risk group (*p* = 5.46e-07), indicating that the risk score was inversely associated with prognosis. Transient receiver operating characteristics (ROC) for 1-, 3-, 5- year in the training set, the area under the curve (AUC) was 0.782 for 1 year, 0.698 for 2 years, and 0.698 for 3 years (Figure 2J), indicating that the signature we established has relatively good predictive performance. In univariate and multivariate analyses, risk score and pathological stage were correlated with OS and were independent predictors of OS (Figures 2K and L). The survival status of each sample and the corresponding risk score are plotted in the training group (Supplementary Figures S2A and B). The expression analysis of the seven genes that make up the prognostic model was carried out in the training group (Supplementary Figure S2C). The results showed that, consistent with the previous conclusion, the genes that make up the model are all high-risk genes were highly expressed in the high-risk group. In addition, further stratified analyses were analyzed to investigate the prognostic significance of features. Our study shows that CR-based signature has better predictive performance in age >65, age ≤ 65, male, grade stage, T stage, pathological stage, M0 stage, and N0 stage (Figure 3). However, due to the lack of patient samples in M ​​and N stages other than M0 and N0 stages, statistical analysis could not be performed. *p* < 0.05 is considered as the standard of significance.

### Clinical correlation analysis and validation of risk score signatures in the ICGC database

**FIGURE 4:**
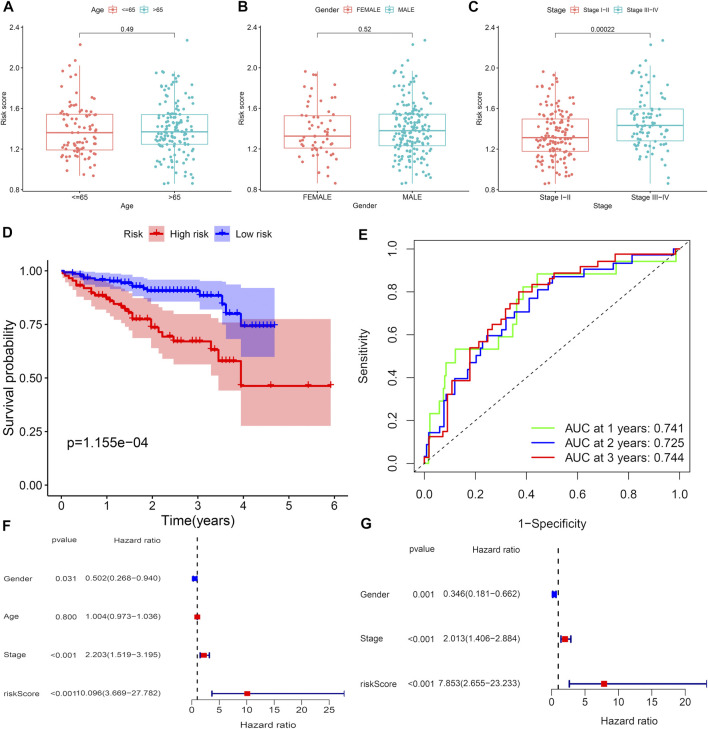
Predictive value of CRs scoring model for the survival status of HCC patients in ICGC queue. **(A–C)** The relationship between risk score and clinicopathological factors, including age **(A)**, gender**(B)**, and pathological stage**(C)**. **(D)** Comparison of total survival time (OS) in low-risk and high-risk groups. **(E)** Sensitivity and specificity of risk scores measured by ROC curves for predicting 1-, 3-, and 5-year overall survival. **(F** and **G)** Forest diagram of univariate and multivariate Cox regression analyses.

The ICGC cohort samples were divided into high-risk groups (*n* = 87) and low-risk groups (*n* = 145) according to the risk score characteristics generated by the TCGA cohort. Furthermore, analysis of the relationship between clinical characteristics (age, gender, and pathological stage) and risk score in the ICGC set. Risk score distribution was not statistically different between risk scores with age and sex of the samples in the ICGC dataset (Figures 4A and B). Higher risk scores were associated with advanced pathological stage (*p* = 0.00022; Figure 4C) and poor overall survival (*p* = 1.155e-04; Figure 4D) in the training set. Figure 4D shows that the high-risk group had a significantly higher mortality rate than the low-risk group (*p* = 1.155e-04), suggesting that the risk score was inversely associated with prognosis, which is consistent with previous conclusions. Transient receiver operating characteristics (ROC) for 1-, 3-, and 5- year in the TCGA set, the area under the curve (AUC) was 0.741 for 1 year, 0.725 for 2 years, and 0.744 for 3 years (Figure 4E), indicating that the signature we established has relatively good predictive performance. In univariate and multivariate analyses, consistent with the previous conclusion in the TCGA set, risk score and pathological stage were independent predictors of OS (Figures 4F and G). The survival status of each sample and the corresponding risk score are plotted in the testing group (Supplementary Figures S2D and E). Expression analysis of the seven genes that make up the prognostic model was carried out in the testing group (Supplementary Figure S2F). The results showed that, consistent with the previous conclusion, the high-risk CRs were highly expressed in the high-risk group.

### Development of a nomogram

**FIGURE 5:**
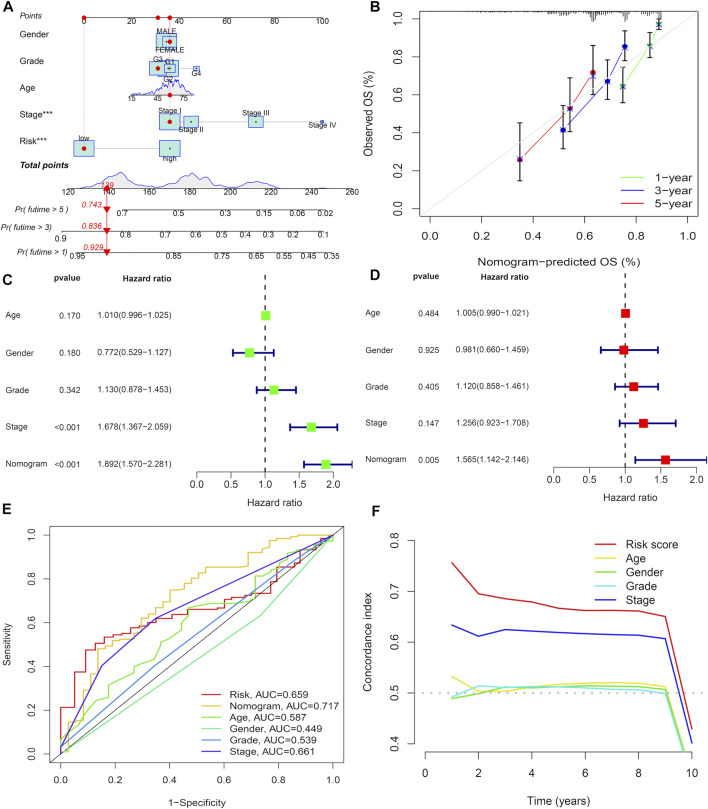
Development of a nomogram. **(A)** Nomogram for predicting 1-,3-, or 5-year OS. **(B)** Calibration plots for predicting1-, 3-, and 5-year OS. **(C)** Univariate Cox regression analysis of the nomogram. **(D)** Multivariate Cox regression analysis of the nomogram. **(E)** ROC curves for CRs score and clinical pathological characteristics. **(F)** C-index of the signature.

For additional prediction of survival in HCC patients, we developed a line map composed of various clinical features, including risk score, pathological stage, grade stage, age, and gender, which effectively predicted the prognosis of HCC patients for 1-, 3-, and 5-years (Figure 5A). Calibration curves demonstrate that nomograms are effective in predicting patient outcomes at 1-, 3-, and 5-years (Figure 5B). Univariate analysis showed that among the factors associated with OS, including pathological stage and nomogram (Figure 5C), multiple regression analysis shows that the survival rate of HCC nomogram is an independent factor for the prognosis of HCC (Figure 5D). The area under ROC curve shows that compared with age, sex, TMN stage, and prognostic risk scoring model, nomogram (AUC = 0.717) has a better prognostic value (Figure 5E). The C-index of signature is higher than other indexes, which proves the favorable forecasting ability of signature (Figure 5F).

### Gene set variation analysis

**FIGURE 6:**
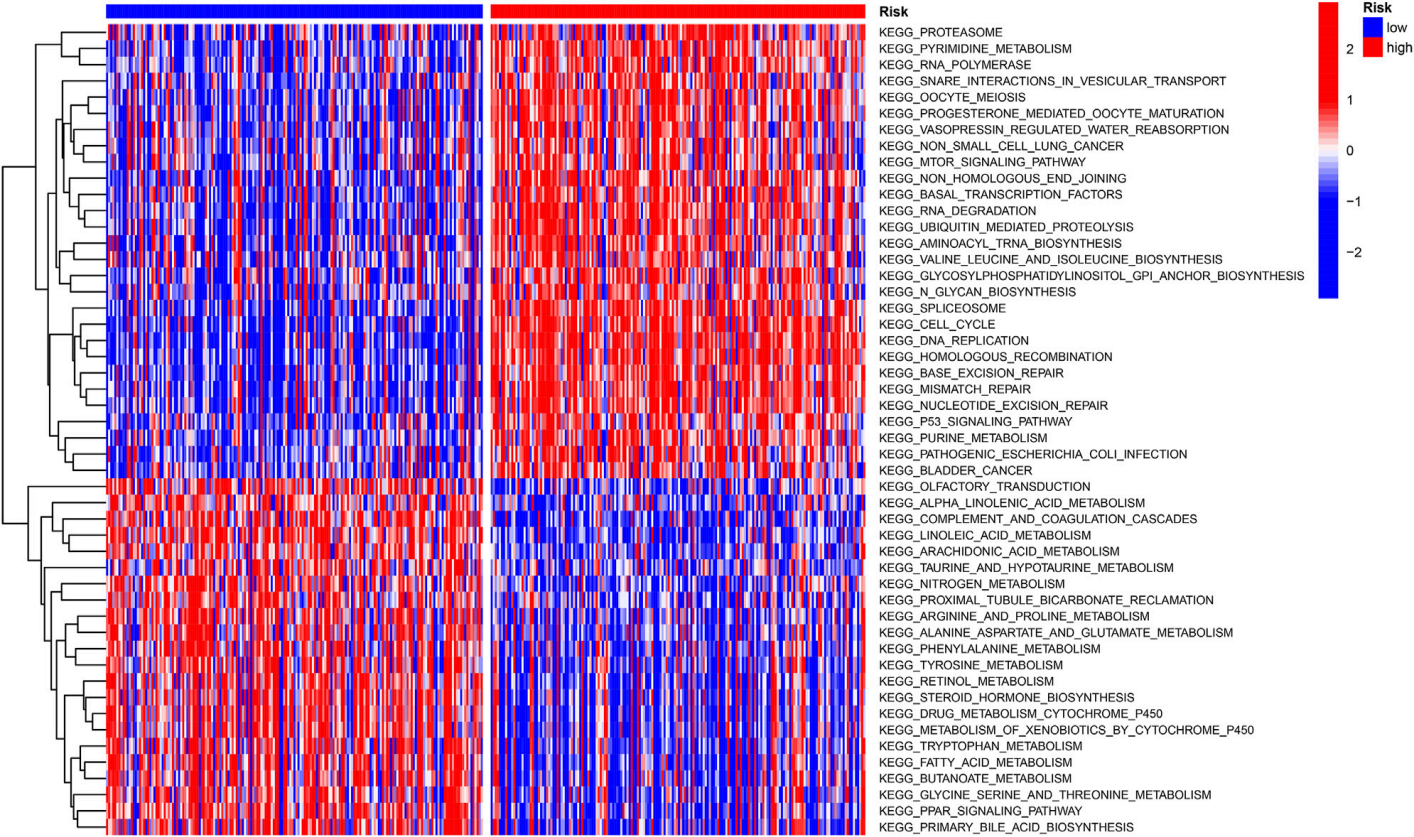
Heatmap of GSVA enrichment between low-risk and high-risk score groups.

The “c2.cp.kegg.v7.4″ gene set downloaded from the database (MSIGDB) was used for GSVA enrichment to explore the biological behavior. Interestingly, many carcinogenic signaling pathways show high-risk scores, such as the P53 signaling pathway and MTOR signaling pathway, which are closely related to the development of HCC. Most metabolic pathways such as fatty acid metabolism, nitrogen metabolism, and arginine and proline metabolism are enriched with low-risk scores (Figure 6).

### Immune-related features of the CRs-based signature

**FIGURE 7:**
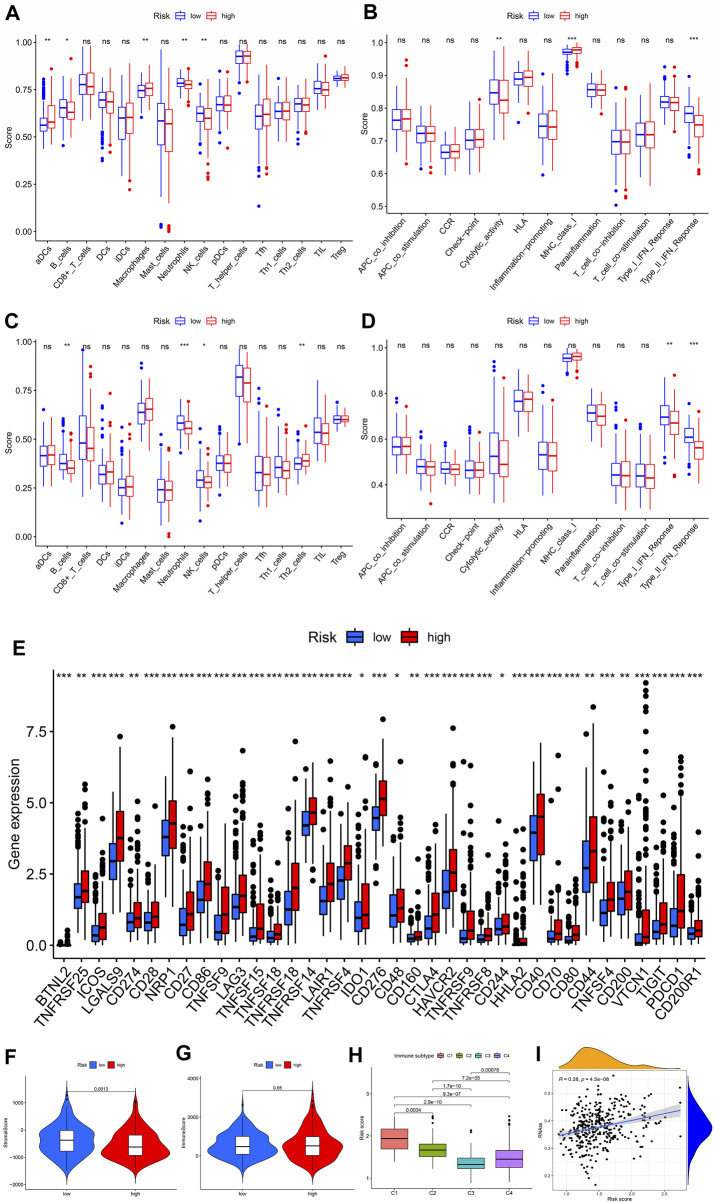
Relationship between CRs model and immunity. **(A** and **B)** Score of 16 immune cells in the TCGA group and ICGC group. **(C** and **D)** Score of 13 immune-related functions in the TCGA group and ICGC group. **(E)** Expression of immune checkpoints in high- and low-risk groups in TCGA queue. **(F** and **G)** Correlation between CRs score and immune and stroma scores. **(H)** Risk score difference in CMS subtypes.

To examine the relationship between risk scores and immune status, we used ssGSEA to assess cumulative scores for different immune cell subsets, associated functions, or signaling pathways. We found that the scores of aDCs, B cells, macrophages, neutrophils, and NK cells were significantly different in the high- and low-risk groups of the TCGA cohort. Among them, aDCs and macrophages have higher scores, while others have lower scores (Figure 7A). Furthermore, the high-risk group activated the type Ⅱ IFN response function and MHC class Ⅰ, and other functions were not activated, indicating that immunosuppressed high-risk groups respond to immunotherapy (Figure 7B). Comparisons in the ICGC cohort confirmed differences in B cells, neutrophils, NK cells, and type II IFN responses between the two risk groups (Figures 7C and D). Furthermore, immune checkpoints play an important role in therapy, and we investigated the correlation between risk scores and key immune checkpoints. We found that almost all immune checkpoints were activated in the high-risk group (Figure 7E), indicating that high-risk groups had immunosuppressive and fatigue phenotypes. High-risk groups exhibited higher stromal scores; however, immune scores did not differ significantly between high-risk and low-risk groups (Figures 7F and G). Furthermore, patients with the CMS1 phenotype had a higher risk score (Figure 7H), suggesting that the CRs-based signature is a novel biomarker for assessing immunotherapy and clinical prognosis. In addition, we assessed the potential correlation between risk score and CSC score, and the results showed that risk score was positively correlated with CSC score, indicating that HCC cells with higher scores had more prominent stem cell characteristics and lower levels of cellular differentiation (Figure 7I). These results suggest that immunotherapy may be more beneficial in high-risk groups.

### Drug sensitivity analysis

**FIGURE 8:**
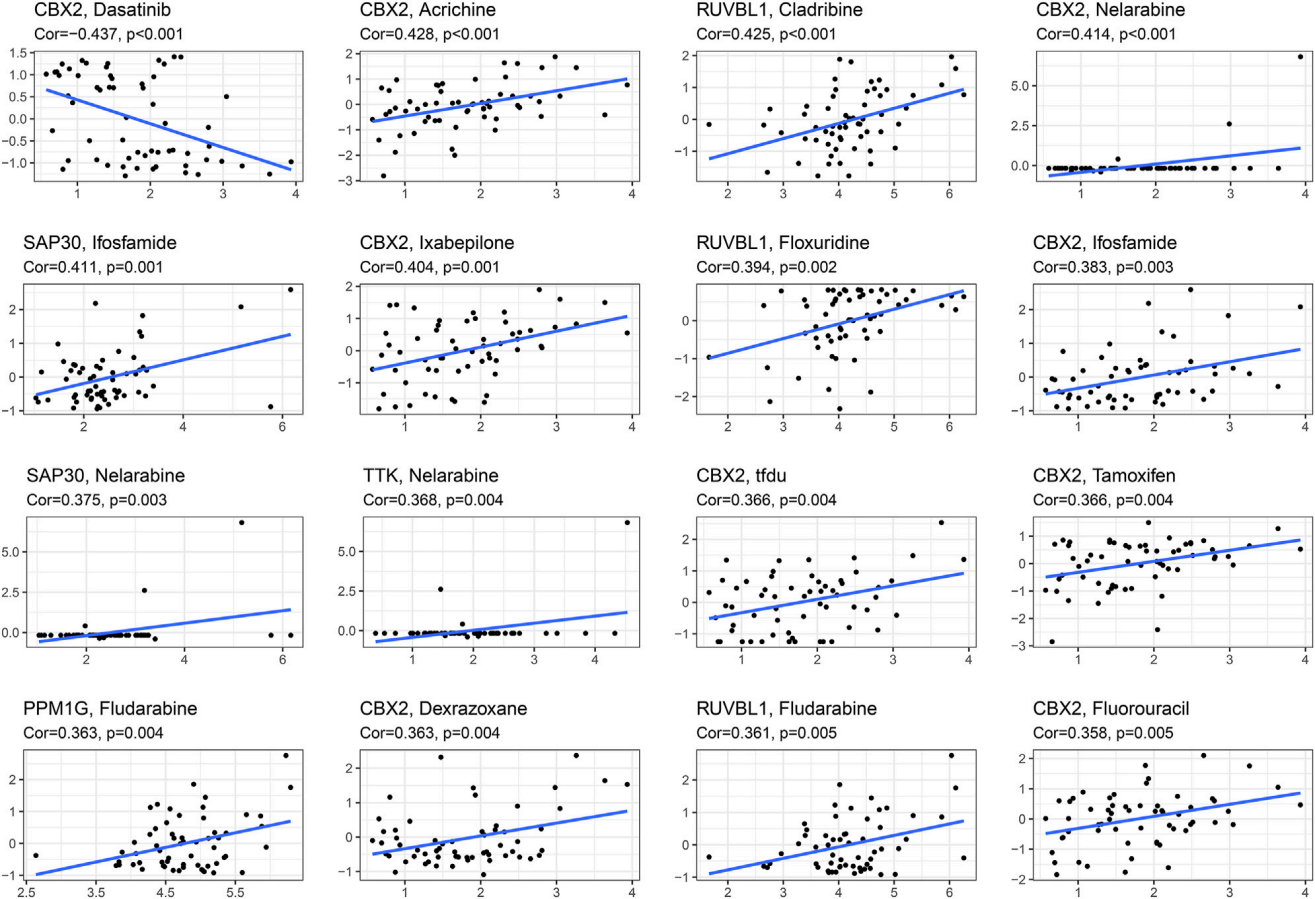
Drug sensitivity analysis based on CellMiner database, screening the first 16 drugs with high correlation with gene expression in CRs prognostic model.

We obtained the top 16 drugs with the largest statistical differences by performing individual sensitivity analyses on the CR that constituted the prognostic model. As the most important part of the results, we found that CBX2 expression was positively correlated with sensitivity to acrichine, nelarabine, ifosfamide, ixabepilone, tfdu, tamoxifen, fluorouracil, and dexrazoxane; however, the expression of CBX2 was negatively correlated with the sensitivity of dasatinib (Figure 8).

## Discussion

Growing research suggests that chromatin regulators play an important role in tumor development. The lack of chromatin regulators ASXL1 activates RAS signaling pathways, accelerating the progress of myeloid malignancies (Zhang, Jiang et al., 2019). CheRNA is a rich blend of RNA regulators in the function of tumors associated with CSC proliferation and dry maintenance (Zhang, Ding et al., 2021). PRC2 mediates the trimethylation of lysine 27 to histone H3, a key factor regulating epigenetic plasticity in gliomas (Natsume, Ito et al., 2013). Previous studies have demonstrated that CRs-based signatures have predictive power for the prognosis of BLCA patients (Zhu, Liu et al., 2022). However, few studies have systematically analyzed the clinical importance of CRs in HCC, and exploring the role of CRs in HCC can guide effective treatment strategies.

This study first examined differentially expressed CRs between normal and tumor tissues and then constructed a prognostic risk score model consisting of seven CRs in the TCGA cohort by univariate Cox regression analysis and LassoCox regression analysis. In the TCGA set and the ICGC set, the overall survival rates of different risk groups were significantly different, suggesting that the prognostic risk assessment model can be used to screen rats with poor survival. In addition, the risk assessment nomogram incorporates some clinicopathological features, further enhancing the clinical utility of this prognostic risk scoring model.

GSVA analysis shows that CR-based characteristics are mainly related to cancer- and metabolism-related pathways, such as the P53 signaling pathway and mTOR signaling pathway. Therefore, the signature based on CRs has the ability to predict the prognosis of HCC patients and may play an important role in HCC biology. P53 haploid insufficiency is helpful for the mTOR signal to pass through PTEN/PI3K/Akt axis and promote HCC tumorigenesis (Luo, Fang et al., 2021). The mutation of p53 in liver cancer may provide a new opportunity for treatment (Muller and Vousden 2014).

Higher risk scores in HCC patients were associated with lower progression-free survival, suggesting that prognostic risk assessment models for chromatin regulators could be used to personalize treatment. Immune checkpoints are effective in high-risk patients who require immunotherapy. Therefore, it is very important to establish an appropriate model to distinguish which patients are suitable for immunotherapy. Research on immune checkpoint inhibitors is booming (Zongyi and Xiaowu 2020). Patients with high-risk scores were more common in aDCs and macrophages. Studies have shown that the increase in tumor-associated macrophages is due to their role in immune invasion, leading to poor prognosis in HCC patients (Zhou, Zhou et al., 2016). Patients with high-risk scores were more common in aDCs and macrophages. Studies have shown that the increase in tumor-associated macrophages is due to their role in immune invasion, leading to poor prognosis in HCC patients.

In conclusion, we created a prognostic marker model consisting of 7 CRs. The TCGC and ICGC databases showed that the model was OS-independent and strongly correlated with the immune microenvironment, tumor microenvironment, and drug sensitivity. It provides new ideas and methods for predicting liver cancer, immunotherapy, and evaluating drug sensitivity. However, there were no pivotal trials or large clinical trials in this study to confirm this result.

## Data Availability

The original contributions presented in the study are included in the article/Supplementary Material; further inquiries can be directed to the corresponding author.

## Abbreviations

HCC, hepatocellular carcinoma; CRs, chromatin regulators; TCGA, The Cancer Genome Atlas; GEO, Gene Expression Omnibus; KEGG, Kyoto Encyclopedia of Genes and Genomes; FDR, false discovery rate; FC, fold change; GO, Gene Ontology; OS, overall survival.
